# Prophylactic Dose of Oxytocin for Uterine Atony during Caesarean Delivery: A Systematic Review

**DOI:** 10.3390/ijerph18095029

**Published:** 2021-05-10

**Authors:** Vilda Baliuliene, Migle Vitartaite, Kestutis Rimaitis

**Affiliations:** 1 Department of Anaesthesiology, Lithuanian University of Health Sciences, Eiveniu str. 2, LT-50009 Kaunas, Lithuania; kestutis.rimaitis@lsmuni.lt; 2 Faculty of Medicine, Medical Academy, Lithuanian University of Health Sciences, A. Mickeviciaus str. 9, LT-44307 Kaunas, Lithuania; migle.vitartaite@stud.lsmu.lt

**Keywords:** oxytocin, uterine contraction, caesarean delivery, hemorrhage

## Abstract

Objective—to overview, compare and generalize results of randomized clinical trials analyzing different oxytocin doses to prevent postpartum hemorrhage, initiate and maintain uterine contraction after Caesarean delivery. Methods—‘PubMed’, ‘EMBASE’, ‘CENTRAL’, and ‘CINAHL’ electronic databases were searched for clinical trials analyzing the effectiveness of different dose of oxytocin given intravenously during surgery for uterine contraction and to reduce postpartum hemorrhage. A systematic review of relevant literature sources was performed. Results—our search revealed 813 literature sources. A total of 15 randomized clinical trials, comparing different doses of oxytocin bolus and infusion used after caesarean delivery have met the selection criteria. Conclusion—oxytocin bolus 0.5–3 UI is considered an effective prophylactic dose. Recommended effective prophylactic oxytocin infusion dose is 7.72 IU/h, but it is unanswered whether we really need a prophylactic infusion of oxytocin if we choose effective bolus dose size and rate. Adverse hemodynamic effects were observed when a 5 UI oxytocin bolus was used. However, topics such as bolus dose size, infusion dose size and requirement as well as bolus injection rate, still remain unanswered. The doses that are recommended in the guidelines of peripartum hemorrhage prophylaxis are not confirmed by randomized controlled double-blind trials and more research should cover this topic.

## 1. Introduction

Early postpartum hemorrhage can occur if oxytocin is not used or if the dose of its bolus or infusion is too small. The American College of Obstetricians and Gynecologist defines early (primary) postpartum hemorrhage as at 1000 mL or more of total blood loss or loss of blood accompanied by signs and symptoms of hypovolemia within 24 h following the delivery, including intrapartum loss [1]. The Royal College of Obstetricians and Gynecologists divides it into minor (500–1000 mL) and major (1000 mL and more) categories. They also define late (secondary) postpartum hemorrhage as abnormal or excessive bleeding later than 24 h post-birth but no later than 12 weeks [2]. Parturient hemorrhage has been the main cause of maternal morbidity and mortality for many years all over the world. Postpartum hemorrhage is one of the most common complications after caesarean delivery, which is an important surgical intervention performed in obstetric practice. This has to be considered because caesarean delivery rates in the developing world countries keep getting higher [3,4]. Minimizing the amount of blood lost during caesarean delivery has a great benefit to decrease postoperative morbidity and to decrease the risks associated with blood transfusions. The routine use of oxytocin correlates with a significant reduction in the occurrence of postpartum hemorrhage (PPH) [5,6,7].

Finding an optimal dose of oxytocin in patients undergoing caesarean delivery is a problem of great importance because it is mandatory to achieve an adequate balance between effective uterine contraction to limit postpartum hemorrhage and oxytocin-induced adverse events.

The aim of this review was to analyze the randomized controlled trials and to find the best and lowest possible prophylactic dose of oxytocin during caesarean delivery (CD).

## 2. Methods

The design of this systematic review of the literature is followed by the Preferred Reporting Items for Systematic Reviews and Meta-Analyses (PRISMA) statement guidelines. Data were identified from searches of MEDLINE (PubMed), Excerpta Medica Database (EMBASE), Cochrane Central Register of Controlled Trials (CENTRAL), Cumulative Index to Nursing and Allied Health Literature (CINAHL) databases and conducted up to the 15 November 2020. The combination of keywords included terms ‘oxytocin’ and ‘caesarean’ and ‘dose’ or ‘hemorrhage’ in PubMed Advanced Search Builder in all fields accordance with the PICO criteria: ‘participants’ were limited to pregnant women, ‘interventions’ covered were randomized controlled clinical trials on caesarean delivery, ‘comparator’—comparing oxytocin with placebo or a different dose of oxytocin, ‘outcomes’ discovered after a thorough analysis of researches and classified into categories according to the trial type and most common findings. Records were screened by the title, abstract and full text by two independent investigators (V.B. and M.V.). Any disagreements were resolved through evaluation and discussion or by consulting a third independent investigator who made the final decision. Inclusion criteria were: (1) full text articles published in English; (2) not older than 2004; (3) single, double or triple-blinded randomized trials of different oxytocin intravenous dosage and/or placebo; (4) caesarean delivery; (5) aged over 18. However, we review or meta-analyze systematic review articles, commentaries, abstract-only publications, guidelines, case reports, trials with oxytocin and carbetocin, other than oxytocin uterotonic agent, but not randomized trials, and dose-ranging trials were excluded.

**Figure 1:**
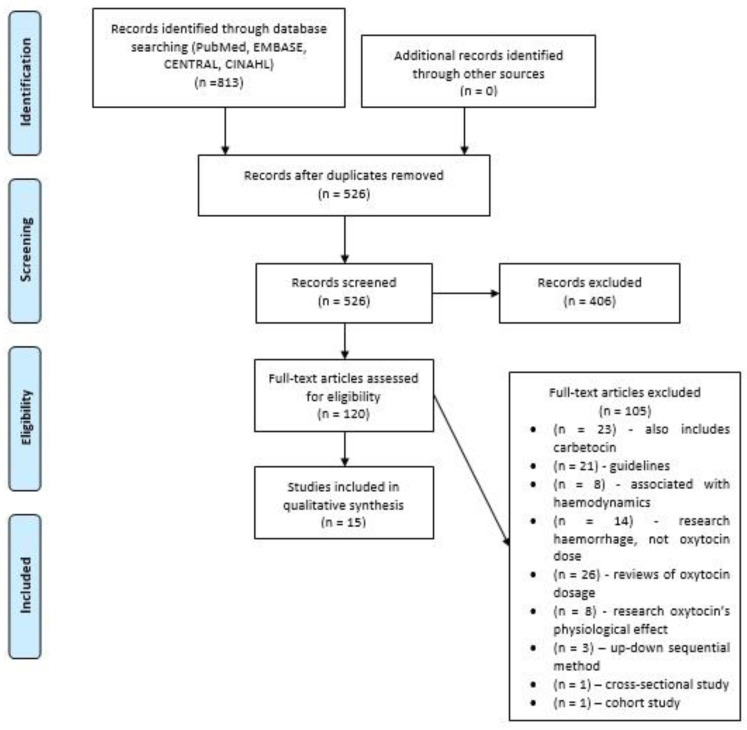
Flow chart

The detailed search flowchart is presented in Figure 1.

## 3. Results

### 3.1. Study Selection Process

The research yielded 813 results, extracted from one database. All duplicates were removed, 526 articles were checked manually for relevance by screening their titles and abstracts. A total 120 results met the inclusion criteria, but only 15 were included after a full-text review. Only full-text articles were selected because the information given in the abstract was not sufficient for the thorough analysis. The randomized trials, conducted in 2008–2020, which compared different doses of oxytocin or oxytocin and placebo given for patients during CD, in order to investigate the effect of the drug on PPH and uterine contraction, were analyzed. A total of 105 publications were excluded for reasons explained in Picture 1.

### 3.2. Characteristics of Included Studies

Those 15 selected trials could be divided into several groups: those that investigate different bolus dose (5 trials), those that investigate different infusion dose (9 trials), and those that investigate both (1 trial). A summary of the results of 15 analyzed studies is provided in Table 1.

### 3.3. Synthesis of Results

All 15 trials (bolus/infusion/both) included in this systematic review were investigated and compared in 4 categories: PPH and blood loss, uterine contraction, and hemodynamics. The summarized results can be seen in Table 2.

Butwick and colleagues compared 75 women divided into 5 groups to receive 0, 0.5, 1, 3, or 5 IU oxytocin bolus intravenously, diluted with 5 mL of 0.9% normal saline and administered over the time period of 15 s. Adequate uterine tonus was measured at 2, 3, 6, and 9 min. There were no significant differences of adequate uterine tonus at 2 min between the groups. Sixty-six per cent of participants in the placebo group had adequate uterine tonus at 3 min and 100% of the parturient receiving 3 IU of oxytocin (*p* = 0.04). This study results indicated that adequate uterine tonus for patients during caesarean delivery can be achieved using a small, 0.5–3 IU bolus of oxytocin plus continuous infusion of 250 mL normal saline with 10 IU of oxytocin [8].

Sartain with co-authors studied data of 80 women, who were given 2 IU or 5 IU oxytocin bolus over 5–10 s and oxytocin infusion of at rate 10 IU/h for 4 h (40 IU totally) after delivery. No difference was found in blood loss, uterine tone, or need for additional uterotonic drugs. Nonetheless, heart rate was found higher, mean arterial pressure was found smaller, and frequency of nausea and antiemetic drugs were bigger in the 5 IU oxytocin bolus group [9].

Somjit and co-authors compared 5 IU and 10 IU oxytocin bolus infused over 15 s and followed by the infusion with 20 IU of oxytocin (2.5 IU/h) impact on uterine contraction. Fewer patients, who received 10 IU oxytocin bolus needed additional uterotonic agents. Blood loss and the uterine tone did not differ significantly. They concluded that 5 IU was non-inferior to 10 IU oxytocin [10].

King with co-authors investigated the impact of 5 IU oxytocin bolus injected over 30 s on women who have at least one risk factor for uterine atony. One hundred and forty-three participants were divided into 2 groups: one group received 5 IU oxytocin bolus followed by 40 IU in 500 mL and 20 IU in 1 L saline oxytocin infusions and the second were given placebo bolus. The number of women requiring an additional dose of uterotonic agents was similar in both groups. They found a significant difference in uterine tone after delivery, which was bigger in the group who received 5 IU oxytocin bolus (*p* < 0.001). However, this result was transient and disappeared after 5 min. Therefore, this study revealed that there is no difference in PPH between women who receive oxytocin bolus and those who do not [11].

Jonsson with colleagues investigated 5 and 10 IU of oxytocin IV bolus, injected over 1 min, impact on electrocardiography. Further doses of uterotonic drugs were available upon request. They found a significant difference in the occurrence of ST depressions associated with oxytocin administration. The conclusion was that 5 IU has less effect on changes in ECG [12].

Kovacheva and colleagues from Boston conducted a study where one group of 30 women received 3 IU oxytocin bolus (over 15 s) and 500 mL saline infusion and the other group of the same size were given placebo bolus and 30 IU oxytocin in 500 mL infusion. Both groups received additional oxytocin according to uterine tone, assessed between 3 min and 12 min, if necessary. Women who received oxytocin bolus also received less oxytocin overall to achieve adequate uterine tone. No differences in hemodynamic or blood loss occurred [13].

Cecilia and colleagues conducted a trial of 271 women randomized into 2 groups: the first group received 10 IU of oxytocin in 500 mL of fluids over 2–4 h, while the second group was given 30 IU of oxytocin in 1500 mL of fluids over 8–12 h. The atonic uterus was seen in 1 patient from the first group and 7 women from the second group (*p* = 0.03). Postoperative fall in blood pressure, tachycardia, and amount of blood loss during the operation and postoperative period and blood transfusions were similar between groups. The conclusion was that a low-dose oxytocin regimen is as effective as a high-dose oxytocin regimen in the prevention of PPH in the postoperative period after caesarean delivery [14].

Ghulmiyyah and colleagues randomly assigned 189 patients into 3 groups where women received 20, 30, or 40 IU of oxytocin in 500 mL solution over 30 min. No significant difference in the change of hemoglobin concentration was observed. This implies that 20 IU oxytocin diluted in 500 mL solution is an appropriate dose for the prevention of PPH [15].

Duffield with colleagues included 51 women: 24 patients received 10 IU of oxytocin in 1 L solution and 27 received 60 IU of oxytocin in 1 L solution. Additional bolus doses of 1 IU oxytocin were given after evaluating uterine contraction between 2 min and 20 min at 2 min intervals if requested. They did not observe any statistically significant difference in excessive blood loss between groups, concluding that both low and high infusion of oxytocin works in the same preventive regime [16].

Gungorduk and co-authors investigated 360 women who were given 5 IU of oxytocin bolus (over 5–10 s) and consecutive infusion with placebo and other 360 participants received 5 IU of oxytocin bolus and consecutive infusion of 30 IU of oxytocin. Mean estimated blood loss (*p* < 0.001) greater than 1000 mL were significantly less in the group of patients who were given 30 IU oxytocin infusion. More women in the placebo group required an additional uterotonic agent (*p* < 0.001) and blood transfusion (*p* = 0.03). This study revealed that 30 IU oxytocin infusion given after bolus lowers the blood loss after CD [17].

Kajendran with other researchers aimed to find out the difference in blood loss between two groups of patients, who were given 5 IU oxytocin bolus over 5–10 and 46 were given 20 IU oxytocin in 500 mL saline, and the other half were given just 500 mL saline later. The blood loss in the group of 20 IU oxytocin infusion was significantly less (*p* = 0.046). Visual estimation of blood loss made by the surgeon (*p* = 0.01) and anesthetist (*p* = 0.03) was also significantly higher in the placebo group [18].

McLeod studied hemodynamic differences between 2 groups of women: both groups were given 5 UI oxytocin bolus over 3 min and 39 patients received 30 UI oxytocin infusion or 35 women were given placebo infusion. There were no significant differences between groups during the 4-h study period. An additional oxytocin infusion after the bolus does not have a significant impact on patient hemodynamic [19].

Murphy with other scientists compared the blood loss of 110 women divided in the same 2 groups as McLeod did. They favored the group with oxytocin infusion because the blood loss was lower, PPH occurred rarely and almost none needed additional uterotonic agent [20].

Sheehan with co-authors conducted a study of 2058 women, who received 5 IU bolus over 1 min, but half of them were given 40 IU oxytocin infusion ant others were given a placebo infusion. No difference was found in PPH between groups (*p* = 0.86). There was a significant difference in uterotonic agent need, it was higher in the placebo group (*p* < 0.001) [21].

Qian and others conducted a trial in 2018 to find out the rate of oxytocin infusion. All 150 women received 1 IU bolus of oxytocin followed by an oxytocin infusion at 0, 1, 3, 5, 8 IU/h. They found a 95% effective dose to be 7.72 IU/h and that the total oxytocin dose administered after delivery could be decreased if an infusion of oxytocin is given [22].

## 4. Discussion

Prophylactic uterotonic agents can prevent PPH and are routinely recommended. A systematic Cochrane review on uterotonic agents, which included 196 clinical trials (135,559 women), and was published in 2018, concluded that oxytocin is effective for preventing peripartum hemorrhage when compared with placebo or no treatment. Ergometrine plus oxytocin combination, misoprostol plus oxytocin combination and carbetocin may have some additional desirable effects compared with oxytocin, but the combination of two drugs is associated with more frequent side effects [23].

The main concern related to this topic is that there is not one opinion on what dosage of the uterotonic drug should be administered to prevent uterine atony and PPH. Despite the fact that oxytocin is used in everyday obstetric practice, it seems that it is used empirically. The guidelines present a wide variety of oxytocin bolus doses and different infusion dose and rate and are based on expert opinion mostly, that means that recommendations are not strong.

The current WHO recommendation, published in 2018, for preventing PPH is 10 IU of intramuscular or intravenous oxytocin for the prevention of PPH for all births. That dose is not related to a high rate of side effects and it also can be divided into a smaller intravenous bolus and an infusion. A rapid intravenous bolus injection must be avoided. One of the important research priorities indicated by WHO is the identification of the optimal regimen of intravenous oxytocin at caesarean section [24].

A study conducted in Canada in 2016–2017 showed that for both high- and low-risk CD participants doctors use 5 IU IV oxytocin bolus, although the range differs from 3 to 10 IU. In addition to bolus, they also use an infusion: 20 IU (range 20–40)/L for low-risk CD and 40 IU (range 20–60)/L for high-risk CD [25]. This study inspired the release of a newly updated guideline in Canada. However, the same recommendation concerning CD remained as in the 2009 version, stating that 100 µg of carbetocin should be given as an IV bolus over 1 min instead of continuous oxytocin infusion [26] The. American College of Obstetricians and Gynecologists also released updated recommendations for postpartum hemorrhage in 2017. But prevention of uterine atony was not discussed separately; the closest recommendation was 10 IU of oxytocin bolus IV [27]. NATA consensus statements on prevention and treatment of postpartum hemorrhage were issued in 2019. They recommended administering an adjusted 5–10 IU intravenous oxytocin dose as the preferred preventive treatment [28]. The Royal College of Obstetricians and Gynecologists in the United Kingdom released updated recommendations for the prevention and management of postpartum hemorrhage in 2016. They recommend the same 5 IU oxytocin dose by slow IV injection [5]. Guidelines released in France in 2015 stated that for prevention of postpartum hemorrhage after CD should be used 5–10 IU of oxytocin dose, injected slowly (at least one-minute) IV [29].

It is very important to remember the fact that oxytocin is used for uterine atony prophylactics for most obstetric patients as supplementation of endogenous oxytocin.

Carvalho dose-finding study results revealed that bolus should be only 0.35 IU, but it should be followed by oxytocin infusion [30].

Balki and co-authors compared 30 women who were given 0.5 IU/mL IV oxytocin after the delivery. If the patient did not respond adequately to the initial bolus of oxytocin, the initial dose for the next patient was increased by 0.5 IU. All patients received 20 IU oxytocin in 1 L saline. All patients who received 3.5 IU initial oxytocin dose showed adequate uterine response within 1–2 min. An important fact is that all parturients were given oxytocin before CD for a minimum of 2 h, so the sensitivity of oxytocin receptors could be reduced. This study indicated that women undergoing CD for labor arrest require approximately 3 IU of oxytocin as a bolus to achieve adequate uterine contraction and continuous oxytocin infusion of 20 IU in 1 L saline after that [31].

These two studies revealed that an adequate dosage of oxytocin bolus can be as small as 3 IU or even less and an additional continuous infusion of oxytocin is needed. The previous oxytocin administration has no influence on the dose.

A cross-sectional study carried out by Beiranvand et al. concluded that the minimum effective dose is 1 IU, and in those in labor progress 1–1.5 IU, both followed by 20 IU oxytocin in 1000 mL infusion [32]. The previously mentioned up-down sequential method study of Carvalho also resulted in a small minimum effective dose.

We analyzed 5 studies investigating superior bolus dose. The four trials comparing constant continuous infusion of oxytocin and different bolus doses [10,11,12,13], two of them had the placebo group [10,13] found that smaller bolus dose or even no bolus dose has the same effect as the bigger one. Sartain concluded that 2 IU is superior to 5 IU especially if hemodynamics was analyzed [11]. In the study of Butwick, more than a half of patients in the placebo group didn’t require a bolus dose [10], which is also confirmed by King, his study revealed that there is no difference in PPH between women who receive oxytocin bolus and those who do not [13]. Jonsson conducted a trial without continuous infusion and also concluded that a smaller dose has lower incidence of cardiovascular side effects [14]. The cardiovascular effect of oxytocin is very important. It is detected that 10 IU oxytocin bolus may cause temporary hypotension and tachycardia as well as myocardial ischemia [33]. Therefore, when comparing the cardiovascular system response to oxytocin dosage, a lower strategic tactic should be approached.

Both Cecilia, Ghulmiyyah, and partly King, investigated the effect of oxytocin infusion without bolus dose. All of them found that the bolus of oxytocin is not necessary to achieve adequate and wanted uterine contraction and prevent PPH and the smaller dose of oxytocin (10–20) given as continuous infusion has the same effect as the bigger one [13,16,17]. This should also be taken into the consideration, as previously discussed, cardiovascular changes are observed almost in every patient given at least 5 IU bolus of oxytocin, so the strategy of using only the infusion of oxytocin could be further researched or bolus dose should be as small as possible.

The inclusion criteria met only one study investigating different bolus doses and continuous infusion of oxytocin. Kovacheva found out that the clinical effect is similar, but additional doses of oxytocin and side effects are lower in the bolus group. The results suggest that prophylactic bolus dose is sufficient [15]. The possibility to avoid the infusion dose helps to reduce the total dose of oxytocin.

Duffield compared low and high doses of oxytocin given as a continuous infusion. That study confirmed the previously mentioned result that both low (10 IU) and high (60 IU) infusion of oxytocin works in the same preventive regime [18].

Although Duffield says that both high and low-dose infusions work in the same way, receptor overstimulation should be taken into the consideration. It is known that clinical use of high-dose of oxytocin infusion longer than 3 to 4 h may diminish the response of uterus tonus to oxytocin [34]. Robinson and colleagues in their study about oxytocin-induced desensitization of the oxytocin receptor concluded that oxytocin-induced desensitization of myocytes to oxytocin stimulation occurred over a clinically relevant time frame (4.2 h). Continued responsiveness of the cells to prostaglandin stimulation after 6 h of oxytocin pre-treatment indicated that post-receptor signaling pathways were maintained, which indicates that the oxytocin receptor is likely involved in the mechanism of myocyte desensitization to oxytocin stimulation [34]. Especially if as high as 60 IU doses of oxytocin were used to prevent PPH. High doses work in the same way as low ones do because receptors cannot bind more hormone molecules to themselves, meaning that there is no actual effect on uterine contraction after infusing oxytocin for too long and too much.

We included five studies that had a very similar design. The bolus dose of oxytocin 5 IU was given and it was followed by infusion with placebo or oxytocin. Oxytocin dose ranged between studies from 20 to 40 IU. The conclusion is that additional oxytocin infusion lowers the risk of PPH, reduces the need for additional uterotonic agents, and does not have a significant impact on patients’ hemodynamic [19,20,21,22,23]. The discussion remains if and when the oxytocin, given as an infusion, is for prophylactic or for treatment of uterine atony because the infused oxytocin dose in mentioned studies is more appropriate for uterine atony treatment. The trials investigating the oxytocin effect on uterine tonus during CD, have superiority over studies conducted in natural delivery because there is a possibility to evaluate real uterine tonus visually and by palpation and to find out the smallest effective dose of the hormone.

One more point for discussion is the duration of oxytocin infusion. It is different and ranges from one hour till 24 h between studies. We should take into account that a long-lasting infusion could reduce the sensitivity of oxytocin receptors as mentioned before and oxytocin could lose its power in the case of real uterine atony. Further, one more unanswered question is what duration of continuous infusion should be considered as prophylactic. It is known that clinical use of high-dose of oxytocin infusion longer than 3 to 4 h may diminish the response of uterus tonus to oxytocin [34].

The only study of Qian discusses prophylactic oxytocin infusion dose. They found a 95% effective dose to be 7.72 IU/h and that the total oxytocin dose administered after delivery can be decreased if the infusion of oxytocin is given [24].

The international consensus statement on the use of uterotonic agents during caesarean section was published in 2019. Researchers from 8 different countries reached an agreement that oxytocin is the first-line drug in both elective and intrapartum CD. For the first one bolus of 1 IU oxytocin and infusion starting at 2.5–7.5 IU/h is recommended. For the latter, 3 IU oxytocin bolus over ≥30 s and infusion starting at 7.5–15 IU/h was decided [35].

Oxytocin given in very high doses, change the mean level of arterial blood pressure in an opposing manner, however, under basal physiological conditions, oxytocin does not contribute to blood pressure maintenance [36]. The hypotensive action of oxytocin, demonstrated in animal models, is believed to be mediated by the direct effect on oxytocin receptors in the heart and the indirect effect of the release of atrial natriuretic peptide, brain natriuretic peptide, and nitric oxide in the cardiovascular tissues [30]. This hormone has widely known cardiovascular side-effects, mostly in decreasing blood pressure by causing peripheral vasodilation, increasing the HR, cases of myocardial ischemia, and arrhythmias. Thomas with his colleagues examined the effect of 5 IU bolus and 5 IU oxytocin infusion over 5 min. Both heart rate and blood pressure were lower in the bolus group, meaning that bolus should be injected slower than it is widely used [37].

Langesaeter et al. with invasive monitoring (LiDCOPlus^®^ monitor) in healthy pregnant women, observed an increase in cardiac index, decreased systemic vascular resistance, and systolic blood pressure (range of 36–62 mmHg) 45 s after oxytocin injection [38]. This same group of authors studied 18 patients with preeclampsia who underwent cesarean section. With the same monitoring as the previous study (LiDCOPlus^®^) connected to the radial artery of patients, the authors found an increased heart rate and decreased systemic vascular resistance and blood pressure in all patients receiving oxytocin (5 IU) after delivery [39]. The hemodynamic instability that can occur during postpartum hemorrhage may not be solely due to hypovolemia, but the association of both hypovolemia and use of oxytocin bolus [40].

This review has several limitations. We could apply more strict inclusion criteria and include only randomized double-blind placebo controlled trials. The meta-analysis would provide more reliable results and conclusions; it’s an option for future work.

Most studies mentioned the rate of bolus injection and it ranges from 5 s till 3 min [10,11,12,13,14,15,19,20,21,23,24]. Further research investigating prophylactic bolus dose infusion rate is necessary. It is possible that the slower bolus infusion rate could reduce the frequency of side effects, or the request of additional doses of uterotonic drugs, or even the necessity of oxytocin prophylactic infusion.

## 5. Conclusions

Oxytocin is the routinely used and effective uterotonic drug, but the unanswered topic still remains the bolus dose size, infusion dose size and requirement and bolus injection rate. The doses that are recommended in the guidelines of peripartum hemorrhage prophylaxis are not confirmed by randomized controlled double-blind trials and are only supported by expert opinion and studies with low evidence rates. Oxytocin bolus 0.5–3 UI is considered an effective prophylactic dose. The effective prophylactic oxytocin infusion dose is 7.72 I U/h. However, it is unanswered whether this prophylactic infusion dose of oxytocin is required if an effective bolus dose, size and rate are chosen. In the cases we use an oxytocin infusion we should separate if it is the prophylactic infusion or the treatment of atony. Adverse hemodynamic effects were observed when a 5 UI oxytocin bolus was used. This must be taken into consideration, especially among female patients with any pre-existing heart conditions. One of the important research priorities is the identification of the optimal regiment of intravenous oxytocin at caesarean section.

## Figures and Tables

**Table 1:** Descriptive characteristics of the different trials which researched oxytocin’s bolus, infusion or both.

Primary Author	Country, Year of Publishing	Trial Type	No. of Patients	Type of Anaesthesia	Inclusion Criteria	Exclusion Criteria	Elective CD
Butwick [8]	USA, 2010	Double-blind, randomized	75	Spinal anesthesia	ASA I or II, age between 18 and 40 yr, singleton pregnancies, and elective CD with a pfannensteil incision, spinal anesthesia	Active labor, ruptured membranes, known drug allergy to oxytocin, multiple gestation, significant obstetric disease, known risk factors for postpartum hemorrhage, inherited or acquired coagulation disorder and thrombocytopenia	Yes
Sartain [9]	Australia, 2008	Double-blind, randomized	80	CSE	Elective caesarean section under regional anesthesia	Patients at increased risk of uterine atony or excessive bleeding (more than two previous Caesarean sections, a history of previous post-partum hemorrhage, known placenta praevia or accreta, twin pregnancy, and polyhydramnios) or cardiovascular instability (pre-eclampsia or essential hypertension)	Yes
Somjit [10]	Thailand, 2020	Double-blind, randomized	155	Spinal anesthesia	Singleton pregnancy, age 18–40 years, 37–41 completed weeks of gestational age, ASA class II and scheduled caesarean delivery under spinal anesthesia	Spinal anesthesia had failed or was inadequate, previous uterine surgery other than caesarean section, high risk of uterine atony (macrosomia, chorioamnionitis, polyhydramnion,, uterine mass) or postpartum hemorrhage (placenta praevia or other placenta disorders, history of postpartum hemorrhage, coagulopathy, thrombocytopenia, or pre-eclampsia), or known allergies to oxytocin	Yes
King [11]	Canada, 2010	Double-blind, randomized	143	Epidural/spinal/CSE/GA/neuraxial + GA	Patients scheduled for elective and or emergency caesarean delivery at a time when an investigator was available were approached	Cardiac disease, hemodynamic instability before commencement of surgery, bleeding disorders, or younger than 19 years, or could not understand or read English	Yes/No
Jonsson [12]	Sweden, 2009	Double-blind, randomized	103	Spinal anesthesia	Elective caesarean section under spinal anesthesia, ≥18 years old	Multiple pregnancy, obesity (body mass index > 35), complications to the pregnancy or nonproficiency in the Swedish language	Yes
Kovacheva [13]	USA, 2015	Double-blind, randomized	60	Spinal anesthesia	ASA I or II, between 18 and 40 yrs of age, with singleton pregnancies, and undergoing an elective caesarean delivery with a pfannenstiel incision and a spinal anesthesia.	Presence of labor, ruptured membranes, maternal or fetal risk factors for uterine atony, previous uterine surgery (except for one previous caesarean delivery with a low-transverse uterine incision), maternal risks for hemorrhage, contraindications to spinal anesthesia or any of the uterotonic agents, and maternal or obstetrician refusal.	Yes
Cecilia [14]	India, 2018	Double-blind, randomized	271	Not known	All the women who underwent elective and emergency CD during the study period were included in the study if they gave informed consent.	Anaemia, placenta previa, abruptio placentae, haemolysis, elevated liver enzymes, and low platelet syndrome, presence of bleeding disorders, intraoperative atony of uterus requiring additional uterotonics or severe intraoperative blood loss requiring blood transfusion, severe fetal distress, previous PPH	Yes
Ghulmiyyah [15]	Lebanon, 2016	Double-blind, randomized	189	Not known	Singleton gestation, elective CD at term with no obstetric or medical complication	Multifetal gestation, hypertensive disorders, chorioamnionitis, suspected macrosomia, polyhydramnios, history of PPH, clotting disorders, antecedent intake of magnesium sulphate, history of uterine fibroids, placenta previa/abruption/accrete or those who were in labor	Yes
Duffield [16]	USA, 2017	Double-blind, randomized	51	Intrathecal anesthesia using a spinal or combined spinal-epidural technique	ASA physical class 2, singleton pregnancies, ≥ 37 weeks’ gestational age, elective CD with a pfannansteil incision, and aged between 18 and 40 yrs.	Patients with significant medical or obstetric disease, active labor or ruptured membranes, placenta previa or other placental disorders, multiple gestation, known uterine abnormalities, and allergies to oxytocin.	Yes
Gungorduk [17]	Turkey, 2010	Double-blind, randomized	720	General anesthesia	Estimated gestational age over 38 weeks and required elective caesarean section	Any risk factor for postpartum hemorrhage, anemia, multiple gestation, antepartum hemorrhage, uterine fibroids, polyhydramnion, emergency CD, a history of uterine atony and postpartum bleeding, current or previous history of significant disease including heart disease, liver, renal disorders or known coagulopathy	Yes
Kajendran [18]	Sri Lanka, 2017	Double-blind, randomized	92	Spinal anesthesia	Pregnant women, who were at term, with singleton pregnancies and had a planned elective caesarean section	Women who were in established labor, had multiple pregnancies, established or suspected cases of chorioamnionitis, both minor and major degree placenta praevia and established or suspected cases of placental abruption, previous history of postpartum hemorrhage and coagulation disorders, and women with a history of or had ultrasonically proven fibroids.	Yes
McLeods [19]	UK, 2010	Double-blind, randomized	74	Spinal anesthesia	Elective caesarean section	Placenta praevia, multiple pregnancy, known bleeding disorder or use of anticoagulant therapy, a history of major obstetric hemorrhage or if the surgeon felt that participation was not appropriate, technical problems in the time leading up to administration of oxytocin.	Yes
Murphy [20]	UK, 2009	Double-blind, randomized	110	Regional anesthesia	Elective lower segment caesarean section	Placenta praevia, multiple pregnancy, known bleeding disorder or use of anti-coagulant therapy, a past history of a major obstetric hemorrhage or if the surgeon felt that participation was not appropriate for any reason.	Yes
Sheehan [21]	Ireland, 2011	Double-blind, randomized	2058	Spinal anesthesia	Healthy women at term (>36 weeks) with singleton pregnancy booked for elective CD	Placenta praevia, thrombocytopenia, coagulopathies, previous major obstetric hemorrhage (>1000 mL), known fibroids, or women who received anticoagulant treatment, did not understand English, younger than 18 years.	Yes
Qian [22]	China, 2020	Triple-blind, randomized	150	Epidural anesthesia	ASA II, aged 18–40 years old, body mass index < 40 kg/m2, singleton pregnancy, ≥37 weeks’ gestation age, elective CDplanned with a pannenstiel incision, and planning epidural anesthesia.	Maternal refusal, emergency CD, active labor, ruptured membranes, pregnancy-induced hypertension, placental abnormalities, multiple gestation, uterine fibroids, history of prior peripartum hemorrhage, coagulation disorders, oxytocin allergy, contraindication to epidural anesthesia, and the need for pharmacological anxiolysis	Yes

USA—United States of America, ASA—American Society of Anesthesiologists, CD—caesarean delivery, CSE—combined spinal-epidural, GA—general anesthesia, UK—United Kingdom.

**Table 2:** Results of studies included in systematic review.

Investigation Object	Primary Author	Placebo or Other Group Treatment	Investigative Group Treatment	Results			
				Uterine Tone	Blood Loss	PPH Incidence	ECG And Hemodynamics
Bolus dose	Butwick [8]	Normal saline bolus IV over 15 s and 10 IU oxytocin in 250 mL 0.9% normal saline over 2 h	0.5/1/3/5 IU IV oxytocin bolus over 15 s and 10 IU oxytocin in 250 mL 0.9% normal saline over 2 h	No difference observed at 2 min between all groups.	No difference observed	No data	Hypotension occurred more often in 5 IU group vs. 0 at 1 min.
Bolus dose	Somjit [10]	5 IU IV oxytocin bolus over 15 s followed by 20 IU oxytocin in 1 L of Ringer’s lactate at over 8 h	10 IU IV oxytocin bolus over 15 s followed by 20 IU oxytocin in 1 L of Ringer’s lactate at over 8 h	No difference observed	No difference observed	0	No difference observed
Bolus dose	King [11]	3 mL normal saline IV over 30 s followed by 40 IU oxytocin in 500 mL of normal saline over 30 min via infusion pump, then a second infusion of 20 IU oxytocin in 1 L normal saline over 8 h	5 IU IV oxytocin bolus over 30 s followed by 40 IU oxytocin in 500 mL of normal saline over 30 min via infusion pump, then a second infusion of 20 IU oxytocin in 1 L normal saline over 8 h	Uterine tone score was bigger in investigative group, but disappeared after 5 min	No difference observed	No data	No difference observed
Bolus dose	Sartain [9]	2 IU IV oxytocin bolus over 5–10 s and 40 IU oxytocin in 1 L of Hartmann’s solution over 4 h	5 IU IV oxytocin bolus over 5–10 s and 40 IU oxytocin in 1 L of Hartmann’s solution over 4 h	No difference observed	No difference observed	No data	After oxytocin bolus increased HR was observed <1 min, at 1 min MAP decreased and was greater in the 5 IU, not 2 IU group. 57.5 per cent of 5 IU group HR increase over 30 beats/min 15 per cent in the same group experienced MAP decrease more than 30 mm Hg
Bolus dose	Jonsson [12]	5 IU IV oxytocin bolus over 1 min followed by 30 IU oxytocin in 500 mL 0.9% saline at a rate sufficient to control uterine atony	10 IU IV oxytocin bolus over 1 min followed by 30 IU oxytocin in 500 mL 0.9% saline at a rate sufficient to control uterine atony	9 women in 5 IU group needed additional uterotonic agent	No difference observed	6 (4 in 5 IU and 2 in 10 IU groups)	Less frequently ST depression and decrease in MAP at 2 min was observed in 5 IU oxytocin group
Infusion dose	Cecilia [14]	30 IU oxytocin IV in 1500 mL IV fluids over 8–12 h	10 IU oxytocin IV in 500 mL of IV fluids over 2–4 h	Atonic uterus in 7 women (2.5 per cent) in 30 IU group	No difference observed	2 (1 in each group)	No difference observed
Infusion dose	Ghulmiyyah [15]	20/30/40 IU IV oxytocin in 500 mL of lactated Ringer solution over 30 min followed by consecutively 30 IU then 20 IU then 10 IU of oxytocin in each 1000 mL of lactated Ringer solution (a total of 3 L) for 24 h postpartum.		No difference observed	No difference observed	0	No difference observed
Infusion dose	Duffield [16]	1 IU IV oxytocin bolus and 10 IU oxytocin in 1000 mL lactated Ringer’s solution for 4 h.	1 IU IV oxytocin bolus and 60 IU oxytocin in 1000 mL lactated Ringer’s solution for 4 h.	No difference observed	No difference observed	8 (4 in each group)	No difference observed
Infusion dose	Gungorduk [17]	5 IU IV oxytocin bolus over 5–10 s and a 500 mL of lactated Ringer’s solution for 4 h.	5 IU IV oxytocin bolus over 5–10 s and a 30 IU oxytocin infusion in 500 mL of lactated Ringer’s solution for 4 h	Placebo group required more frequent additional uterotonic agent	The amount of lost blood smaller in the investigative group. Placebo group required more frequent blood transfusion	46 in placebo group and 18 in oxytocin group (12.8 and 5 per cent respectively)	No difference observed
Infusion dose	Kajendran [18]	5 IU IV oxytocin bolus over 5–10 s and 500 mL of 0.9% normal saline for 4 h	5 IU IV oxytocin bolus over 5–10 s and 20 IU oxytocin in 500 mL 0.9% normal saline solution for 4 h	No need for additional uterotonic agent in both groups	Investigative group amount of blood loss was smaller	No data	No data
Infusion dose	Murphy [20]	5 IU IV oxytocin bolus and 500 mL of Hartmann’s solution for 4 h	5 IU IV oxytocin bolus and 30 IU oxytocin in 500 mL of Hartmann’s solution for 4 h	Investigative group—almost none needed additional uterotonic agent	Investigative group amount of lost blood was lower	3 (2 in placebo and 1 in oxytocin group)	No data
Infusion dose	McLeods [19]	5 IU IV oxytocin bolus over 3 min and a placebo infusion of Hartmann’s solution 500 mL over 4 h.	5 IU IV oxytocin bolus over 3 min and a 30 IU oxytocin infusion in 500 mL	No data	No data	No data	No difference observed
Infusion dose	Sheehan [21]	5 IU oxytocin IV bolus over 1 min and 500 mL of 0.9% saline IV over 4 h	5 IU oxytocin IV bolus over 1 min and 40 IU oxytocin in 500 mL 0.9% saline solution IV over 4 h	Placebo group—more frequently needed additional uterotonic agent	No difference observed	317 (159 in bolus group and 158 in bolus and infusion group)	No data
Infusion dose	Qian [22]	1 IU IV oxytocin bolus over 15 s and 50 mL normal saline over 1 h	1 IU IV oxytocin bolus over 15 s and 1/2/3/5/8 IU oxytocin in 50 mL normal saline over 1 h	3,5,8 IU oxytocin groups required rescue oxytocin bolus or uterotonic agent	No difference observed	0	No difference observed
Bolus dose, infusion dose	Kovacheva [13]	3 mL of 0.9% saline bolus over 15 s and 30 IU oxytocin in 500 mL 0.9% saline, wide-open infusion flow rate	3 IU in 3 mL IV oxytocin bolus over 15 s and 500 mL 0.9% saline, wide-open infusion flow rate	Investigative group required less additional oxytocin	No difference observed	No data	No difference observed

IU—international unit, IV—intravenous, HR—heart rate, MAP—mean arterial pressure.

## Data Availability

No additional data available.
